# CO_2_ and N_2_O Emissions From Vehicles in Seoul Megacity, South Korea: Insights From Mixing Ratio and Stable Isotope Ratios

**DOI:** 10.1002/rcm.70094

**Published:** 2026-07-20

**Authors:** Jeongeun Kim, Jinho Ahn, Sambit Ghosh, Sakae Toyoda, Shinji Morimoto, Hyunsuk Choi, Jaehoen Jung, Hungyu Lee

**Affiliations:** ^1^ School of Earth and Environmental Science Seoul National University Seoul South Korea; ^2^ Center for Cryospheric Sciences Seoul National University Siheung South Korea; ^3^ Department of Geology and Geophysics Indian Institute of Technology Kharagpur Kharagpur India; ^4^ School of Materials and Chemical Technology Institute of Science Tokyo Yokohama Japan; ^5^ Center for Atmospheric and Oceanic Studies, Graduate School of Science Tohoku University Sendai Japan; ^6^ Department of Atmospheric Environment Research Seoul Metropolitan Government Research Institute of Public Health and Environment Seoul South Korea

**Keywords:** emission ratio, nitrous oxide, stable isotopes, urban greenhouse gases, vehicle emissions

## Abstract

**Rationale:**

Vehicular traffic is a major source of anthropogenic greenhouse gases in megacities; however, real‐world constraints on vehicle‐derived N_2_O emissions and their isotopic characteristics remain limited.

**Methods:**

Air samples were collected from three tunnels and urban background sites (a university campus and mountain) in Seoul, South Korea. The CO_2_ and N_2_O mixing ratios and isotopic compositions (δ^13^C, δ^18^O of CO_2_; δ^15^N^bulk^, δ^18^O, and site preference of N_2_O) were measured by gas chromatography and isotope ratio mass spectrometry, respectively. The vehicle‐emitted isotopic endmembers were derived using Keeling plot analysis.

**Results:**

The vehicular N_2_O:CO_2_ molar ratio was (4.8 ± 1.9) × 10^−2^ (ppb:ppm). Scaling this ratio to Seoul's on‐road CO_2_ inventory yields an estimated annual N_2_O emission of 163 ± 66 kt CO_2_ equivalents, more than twice the current bottom‐up estimate. Vehicle‐emitted CO_2_ exhibited isotopically homogeneous fossil fuel combustion δ^13^C signatures (−27.6‰ ± 0.4‰) and δ^18^O values (26.8‰ ± 0.5‰), and vehicular N_2_O was characterized by low δ^15^N^bulk^ values (−6.5‰ ± 2.3‰), δ^18^O values (34.1‰ ± 2.2‰), and near‐zero site preference (0.2‰ ± 7.3‰).

**Conclusions:**

The integration of tunnel and tailpipe measurements with stable isotope analyses enables the direct quantification of greenhouse gas emissions from vehicles under real‐world conditions. Vehicle‐derived CO_2_ exhibits a narrow and well‐constrained δ^13^C signature, which is consistent with gasoline‐ and diesel‐dominated fleets in Seoul, whereas N_2_O isotopes reflect the formation processes associated with high‐temperature combustion and catalytic after treatment. Furthermore, systematically lower δ^18^O values in vehicle‐derived CO_2_ and N_2_O relative to the urban background identify a distinct oxygen‐isotope fingerprint of traffic emissions, thereby establishing δ^18^O as a complementary tracer to traffic‐related greenhouse gas quantification.

## 1 Introduction

Fossil fuel combustion from vehicles is a leading source of anthropogenic greenhouse gas (GHG) emissions in megacities, reaching record levels in 2023 [1, 2, 3]. Carbon dioxide (CO_2_) is the primary product of fossil fuel combustion in vehicles, while other GHGs such as methane (CH_4_) and nitrous oxide (N_2_O) also contribute to atmospheric chemistry and climate [4]. CO_2_ isotope ratios (δ^13^C and δ ^18^O) are useful for source apportionment based on atmospheric observations [5, 6, 7, 8, 9, 10]. The δ^13^C values of vehicle‐emitted CO_2_ vary depending on the fuel type because most fossil carbon is oxidized to CO_2_ during combustion [11, 12, 13, 14]. In addition, δ^18^O generally reflects atmospheric O_2_ without significant fractionation [15, 16]; however, during combustion, enrichment by 6‰–9‰ can occur through interactions with ambient moisture [9, 17, 18].

N_2_O is also emitted from vehicles, primarily through three‐way catalytic converters during fuel combustion, with emissions strongly influenced by catalyst operating temperature (maximum at 250°C–400°C) [19, 20, 21, 22]. Transportation N_2_O emissions account for only about 3% of the total anthropogenic N_2_O (0.14 ± 0.07 Tg N yr^−1^ in 2010) [23] but represent the second largest anthropogenic source after agriculture [24]. These estimates are associated with significant uncertainty because they rely largely on studies with limited data coverage. Moreover, N_2_O is a potent GHG, with a global warming potential 273 times that of CO_2_ over a 100‐year time horizon [4] and contributes to stratospheric ozone depletion [25]. Previous studies have shown that high‐temperature combustion and catalytic processes can modify N_2_O isotopic signatures (δ^15^N^bulk^, δ^18^O, and SP) in vehicle exhaust; however, corresponding field‐based constraints for modern urban fleets remain limited [26, 27, 28].

Tunnel studies provide an effective approach for quantifying on‐road vehicle emission ratios by isolating vehicle emissions. Early tunnel studies reported relatively high N_2_O: CO_2_ molar ratios, whereas more recent studies indicate substantially lower values, reflecting advances in emission‐control technologies (Table 1) [14, 20, 28]. Beyond technological progress, real‐world variability in on‐road N_2_O:CO_2_ emission ratios can also arise from differences in vehicle fleet composition, catalyst aging, fuel type, combustion temperature, traffic conditions, road, and operating environments [23, 27, 29, 30].

**TABLE 1:** Comparison of vehicle N_2_O:CO_2_ emission ratios with previous tunnel studies.

References	N_2_O:CO_2_ ×10^−2^ (ppb:ppm)	Location	Measurement year
Berges et al. [20]	14 ± 9	Klara Tunnel, Sweden	1992
Berges et al. [20]	6 ± 3	Elbtunnel, Germany	1992
Becker et al. [19]	4.1 ± 1.2	Kiesberg Tunnel, Germany	1997
Popa et al. [14]	1.8 ± 0.2	Islisberg Tunnel, Switzerland	2011
Laskar et al. [28]	2.3 ± 0.6	Hsuehshan Tunnel, Taiwan	2017
**This study**	**4.8** **±** **1.9**	**Sangdo, Bongcheon, and Gwanak tunnels, South Korea**	**2021–2024**

*Note:* The bold text shows the results from this study.

In South Korea, total greenhouse gas emissions reached 723 Mt. CO_2_ eq. in 2022, with the energy sector accounting for nearly 80% [31]. Transportation contributed approximately 15% of national emissions. In Seoul, this contribution is even more pronounced, as transportation and building‐related energy use together account for nearly 90% of the city's total GHG emissions. Seoul's on‐road vehicle fleet, dominated by gasoline and diesel passenger vehicles with widespread operation of CNG‐powered buses (Table S2), provides a suitable framework for constraining fleet‐integrated vehicle emission signatures under mixed‐fuel conditions [31, 32]. Although Seoul accommodates nearly 20% of the national population within only 0.6% of the country's land area, direct measurements of traffic‐related GHGs remain limited. National CO_2_ emissions are estimated using Tier 2 or 3 country‐specific emission factors, whereas N_2_O inventories still rely on default Tier 1 values from the IPCC report [4], highlighting the need for localized and source‐specific data.

In this study, we used tunnel observations to quantify on‐road vehicle emissions of CO_2_ and N_2_O in the Seoul megacity and constrain their source signatures through combined mixing ratio measurements and stable isotope analyses. Fleet‐averaged N_2_O: CO_2_ emission ratios are used as a basis for assessing vehicle‐related N_2_O emissions against urban inventory estimates, while stable isotope ratios of CO_2_ and N_2_O are analyzed independently to characterize emission processes and source signatures associated with on‐road vehicles.

## 2 Methods

### 2.1 Site Description

**FIGURE 1:**
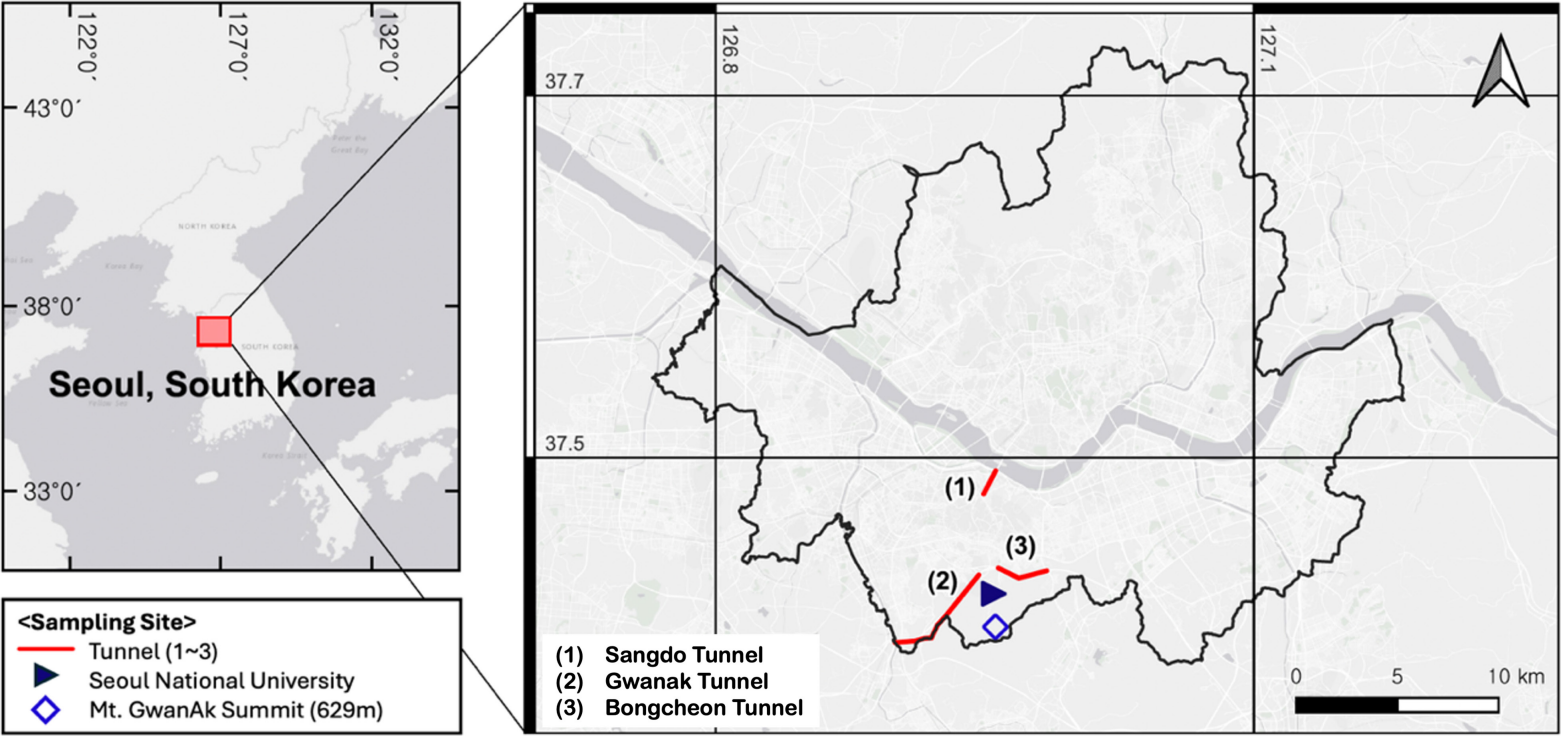
Sampling sites in Seoul, South Korea. Three tunnels were monitored: (1) Sangdo (urban road), (2) Gwanak and (3) Bongcheon (highway). The blue triangle and diamond indicate Seoul National University and Mt. Gwanak summit, representing background air. Sangdo tunnel experiences stop‐and‐go traffic (~20 km/h) due to traffic signals, while Gwanak and Bongcheon tunnels have smoother flow (~80 km/h) with heavy truck and motorcycle restrictions.

Air sampling was conducted in vehicle tunnels and selected ambient sites in Seoul (Figure 1). Three tunnels were investigated: Sangdo (547 m, no ventilation), Bongcheon (2237 m), and Gwanak (4834 m, equipped with ceiling jet fans near the exits). All tunnels consist of two separate bores, each serving a single direction of traffic flow. Camera‐based observations indicated that the vehicle fuel‐type distribution at Sangdo closely matched that of the registered Seoul fleet (2021–2024), and thus this site is considered representative of urban traffic conditions. Bongcheon and Gwanak tunnels are located on express roads where heavy‐duty trucks (over 10 tons) and motorcycles are prohibited, representative of urban on‐road environments.

Ambient air samples were collected at Seoul National University (SNU) Gwanak campus and the summit of Mt. Gwanak (629 m a.s.l) to provide background references and to correct dilution effect in tailpipe samples.

### 2.2 Air Sampling Method

Air samples were collected in 3 L Pyrex flasks (O‐ring stopcocks) and Silcocan canisters (RESTEK), pre‐evacuated overnight and filled with ultra‐high purity N_2_ (> 99.9999%). Air was drawn at 5 L/min using a portable pump through aluminum‐coated tubing (NITTA) equipped with a dust filter and Mg (ClO_4_)_2_ trap, with each container flushed (approximately 10 volumes) and pressurized to 2. To minimize N_2_O artifacts from reactions with NOx, H_2_O, and SO_2_ [33], sample concentrations were analyzed within 1 week of collection.

Tunnel air sampling was conducted during weekday evening rush hours (17:00–19:00) on randomly selected days between 2021 and 2024. Entrance and exit air at Sangdo were sampled simultaneously, whereas sampling at Bongcheon and Gwanak was performed sequentially (~5 min apart) using inlet tubing mounted on an electric vehicle. Ambient samples from Mt. Gwanak were collected in summer (June) and winter (December) 2023, and SNU campus samples were taken between September 2020 and February 2021.

Tailpipe samples were collected from gasoline‐, diesel‐, and CNG‐fueled vehicles using a funnel directly connected to the exhaust under idling and elevated engine speed (1500–2000 rpm) conditions.

### 2.3 Greenhouse Gas Analyses

#### 2.3.1 Mixing Ratio of Greenhouse Gas

The CO_2_ and N_2_O mixing ratios were measured using gas chromatographs (Agilent) equipped with a flame ionization detector (FID) for CO_2_ (via CH_4_ conversion) and an electron capture detector (ECD) for N_2_O [34, 35, 36]. The instruments were calibrated using NOAA‐certified standards (CO_2_: 402.41 ppm; N_2_O: 325.6 ppb), yielding measurement precisions of 0.15 ppm and 0.2 ppb, respectively. Triplicate measurements agreed within 0.25 ppm for CO_2_ and 0.3 ppb for N_2_O. After NOAA‐standard calibration, a system‐specific offset was quantified using dried firn air with independently calibrated concentrations introduced via the sample inlet, and this offset was uniformly applied to all samples. The instrumental drift remained within 0.5 ppm for CO_2_ and 1 ppb for N_2_O.

For interlaboratory comparison, three tunnel samples were analyzed at the Tohoku University [37, 38, 39]. CO_2_ was measured using a nondispersive infrared analyzer (NDIR, precision: 0.02 ppm), calibrated against TU‐2010 working standards (449.13, 409.51, and 421.61 ppm), which align with the WMO X2019 scale [40]. N_2_O was measured by GC‐ECD (Agilent 6890; precision 0.3 ppb) using working standards of 341.24, 321.07, and 370.16 ppb calibrated on the Tohoku University scale, which is 0.5 ppb higher than the WMO scale [41]. All data presented in this study are based on measurements conducted at Seoul National University, while analyses at Tohoku University were performed solely for inter‐laboratory comparison; sample‐specific measurement locations are provided in the Data S1.

#### 2.3.2 Stable Isotope Ratios

The stable isotopes of CO_2_ (δ^13^C and δ^18^O) were analyzed at Tohoku University using a gas isotope ratio mass spectrometer (Finnigan, MAT‐deltaS), following cryogenic CO_2_ extraction into a 6 mm Pyrex tube [40]. The analytical precision was within 0.02‰ for δ^13^C and 0.05‰ for δ^18^O [40, 42]. The final CO_2_ isotopic values were corrected for N_2_O interference, as described in Section 2.3.3 [43, 44].

N_2_O stable isotopes (δ^15^N^bulk^ and δ^18^O) were first measured at Seoul National University using a continuous‐flow IRMS (Delta V Plus, Thermo Fisher) coupled with a PreCon and Conflo IV interface and helium (99.9999%) was used as the carrier gas. Air from a 3 L flask was transferred to 100 mL flasks via a vacuum line, dried using a cold ethanol trap (< −80°C) during the initial 15 min, and the sample was stabilized for 2 h to minimize isotopic fractionation. Following moisture removal, CO_2_ was removed using Mg (ClO_4_)_2_ and ascarite traps, after which N_2_O was cryogenically preconcentrated in the PreCon system, separated on a PoraPLOT Q GC column (35°C), and analyzed for m/z 44, 45, and 46. Isotopic ratios were calibrated against ultra‐pure N_2_O working gas, with analytical precision evaluated using NOAA‐calibrated standard (271.53 ppb), yielding triplicate precisions of 0.10‰ for δ^15^N^bulk^ and 0.17‰ for δ^18^O relative to Air N_2_ and VSMOW scale, respectively.

Selected samples were reanalyzed at the Institute of Science Tokyo (Science Tokyo) to assess measurement reproducibility and container consistency, including δ^15^N^bulk^, δ^18^O, and site preference (SP) (Figure S2, Table S5). SP was determined exclusively at SNU, using an automated GC‐IRMS system (MAT 252, Thermo Fisher) with analytical precisions of 0.1‰ for δ^15^N^bulk^, 0.2‰ for δ^18^O, and 0.5‰ for SP [45].

#### 2.3.3 Calculation of Stable Isotopic Compositions

Stable isotopic compositions are reported in the following δ notation relative to VPDB for carbon, air N_2_ for nitrogen, and VSMOW for oxygen.
(1)

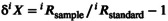




In Equation (1), ^*i*^ *X* denotes the minor isotope of the element in question, and ^*i*^ *R* represents the abundance ratio of the minor isotope to major isotope.

CO_2_ isotopic ratios were corrected for N_2_O interference [40] using instrument‐specific correction factors determined for MAT‐Delta S at Tohoku University (△δ^13^C = 254𝜌 ‰; △δ^18^O = 358𝜌 ‰, where 𝜌 represents the N_2_O to CO_2_ molar ratio). δ^18^O values of CO_2_ reported on the VPDB scale were converted to the VSMOW scale using a constant offset of +41.5‰ to enable comparison with previous studies [14, 46, 47].

For N_2_O, isotope ratios for bulk nitrogen (δ^15^N^bulk^), central nitrogen in NNO molecule (δ^15^N^α^), and oxygen (δ^18^O) were determined, and site preference (SP) was calculated as the difference between δ^15^N^α^ and δ^15^N^β^ (isotope ratios for terminal nitrogen) following standard definitions as follows [48, 49].

(2) $$\delta^{15}N^{\text{bulk}}=\left(\delta^{15}N^{\alpha }+\delta^{15}N^{\beta }\right)/2.$$

(3) $$\mathrm{SP}=\delta^{15}N^{\alpha }–\delta^{15}N^{\beta }.$$

SP is widely used as a process‐specific tracer of N_2_O formation pathways, as it is largely independent of the isotopic composition of precursor nitrogen sources.

At SNU, N_2_O stable isotopic (δ^15^N^bulk^ and δ^18^O) measurements were calibrated using a laboratory working gas (NOAA‐certified) that had been previously calibrated against international standards (Air N_2_ and VSMOW) at Science Tokyo. This working gas was measured alongside tank air to determine the tank reference value (δ_Tank‐Std_). For each analytical session, at least three tank air measurements bracketed the samples, allowing correction for instrumental drift and calculation of δ_Sample‐Tank_. Final sample isotopic compositions (δ_Sample‐Std_) were calculated as follows:

(4) $$\delta_{\text{Sample}-\mathrm{STD}}=\delta_{\text{Sample}-\text{Tank}}+\delta_{\text{Tank}-\mathrm{STD}}+\delta_{\text{Sample}-\text{Tank}}\times \delta_{\text{Tank}-\text{STD}}$$

**FIGURE 2:**
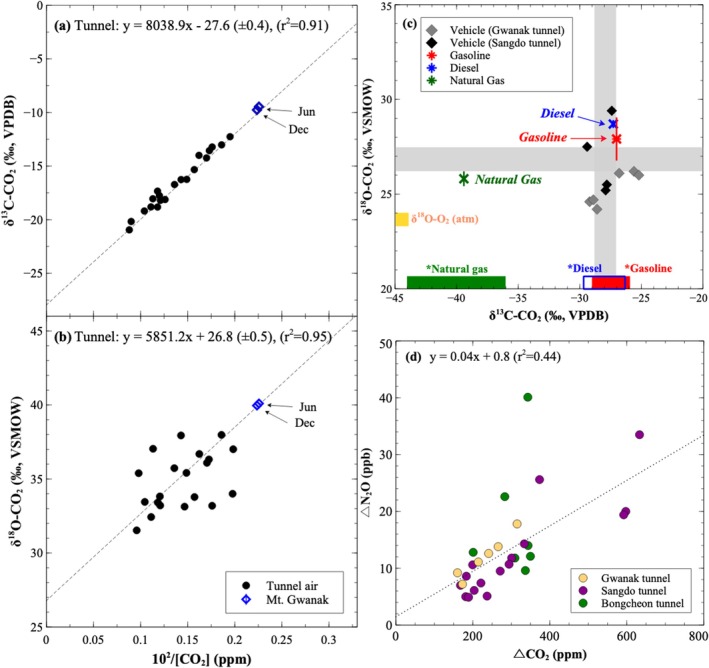
CO_2_ isotopic composition and N_2_O:CO_2_ emission ratio from tunnel and background air samples. (a,b) Keeling plots of δ^13^C and δ^18^O in tunnel air (black circles) and Mt. Gwanak background air (open navy diamonds). (c) Vehicle‐emitted CO_2_ isotopic endmembers from Keeling plot analysis (shaded gray), mass balance (black and gray diamonds), and direct tailpipe measurements (colored diamonds). *Horizontal and vertical reference bars indicate literature‐reported fuel‐specific δ^13^C‐CO_2_ ranges [8, 50, 51, 52, 53] and atmospheric δ^18^O of O_2_, respectively [54, 55]. (d) Relationship between ΔCO_2_ and ΔN_2_O used for N_2_O:CO_2_ emission ratio calculations.

**FIGURE 3:**
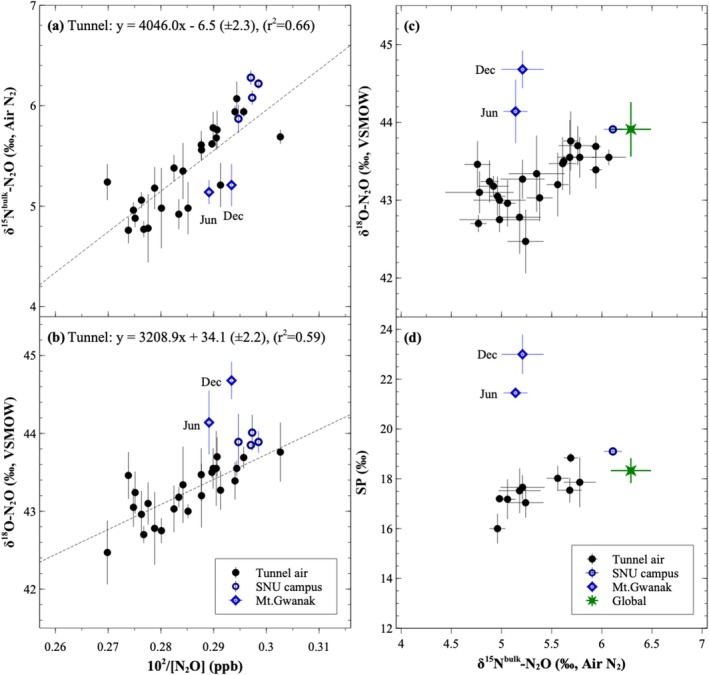
N_2_O isotopic composition in tunnel, urban, and background air. (a,b) Keeling plots of δ^15^N^bulk^ and δ^18^O in tunnel air (black circles), SNU campus (open navy circles), and Mt. Gwanak (open navy diamonds). (c,d) Comparison of δ^15^N^bulk^, δ^18^O, and site preference (SP) among sites, including global background values (green stars) from Ghosh et al. [56], estimated using a Monte Carlo inversion and gradient‐based method.

Vehicle‐emitted CO_2_ and N_2_O isotopic source signatures were derived using Keeling plot analysis, with results independently validated using an entrance‐exit mass balance approach (Figures 2 and 3) [57, 58]:

(5) $$C_{\mathrm{ent}}\times {\mathrm{\delta X}}_{\mathrm{ent}}+C_{v\text{ehicle}}\times {\mathrm{\delta X}}_{\text{vehicle}}=C_{\text{exit}}\times {\mathrm{\delta X}}_{\text{exit}},$$

where *C*_ent_ and *C*_exit_ are the molar ratios, and δX_ent_ and δX_exit_ are the corresponding isotope ratios at the tunnel entrance and exit, respectively. Uncertainties in the Keeling plot‐derived endmembers were estimated using a Monte Carlo approach (*n* = 50 000), incorporating analytical precision of both concentration and isotopic measurements. Concentration uncertainties were propagated in inverse concentration (1/[CO_2_] or 1/[N_2_O]). Sample variability was included by bootstrap resampling (with replacement) of the original data points. Endmember values were defined as the mean ± 1𝜎 of the intercept distribution. Detailed Monte Carlo results are provided in the Data S3.

To facilitate comparison with global atmospheric backgrounds, reference N_2_O isotopic baseline values for 2021 were estimated by extrapolating previously reported global observations, with calculation details provided in Table S6 and Data S4 [56].

## 3 Results

### 3.1 Greenhouse Gas Molar Ratio (N_2_O:CO_2_)

Vehicle‐derived N_2_O:CO_2_ molar ratios were determined from 29 paired flask samples collected at the entrances and exits of three road tunnels in Seoul (Figure 2d). For all sampling events, both CO_2_ and N_2_O molar fractions were consistently higher at tunnel exits than at entrances, indicating the presence of on‐road vehicle emission signals (Figure S1). Vehicle‐related contributions were estimated using entrance‐exit differences in tunnel air, following ∆X = [X]_exit_ − [X]_entrance_ (e.g., ∆CO_2_ = [CO_2_]_exit_ − [CO_2_]_entrance_). The corresponding mean differences were 290.8 ± 129.0 ppm for CO_2_ and 13.7 ± 8.2 ppb for N_2_O (1𝝈). Using these differences, the resulting ∆N_2_O: ∆CO_2_ molar emission ratio averaged (4.8 ± 1.9) × 10^−2^ (ppb:ppm). This value represents fleet‐averaged N_2_O emissions relative to CO_2_ from on‐road vehicles under real‐world driving conditions in Seoul.

### 3.2 CO_2_ Stable Isotope Ratios

The stable isotopic ratios of CO_2_ in the tunnel air ranged from −21.0‰ to −12.3‰ (VPDB) for δ^13^C and from 31.5‰ to 38.0‰ (VSMOW) for δ^18^O (Figure 2a,b). Both isotopic ratios exhibited negative correlations with CO_2_ mixing ratios, associated with lower δ^13^C and δ^18^O values with higher CO_2_ concentrations. Keeling plot analyses of air samples from the Sangdo and Gwanak tunnels yielded vehicle‐emitted CO_2_ isotopic endmembers of −27.6‰ ± 0.4‰ for δ^13^C and 26.8‰ ± 0.5‰ for δ^18^O, based on Monte Carlo‐derived uncertainties (1𝜎). The mean endmember values did not differ significantly between the Sangdo and Gwanak tunnels (*p* = 0.40 and 0.20 for δ^13^C and δ^18^O, respectively). Direct tailpipe exhaust isotope measurements were corrected for atmospheric dilution using mass balance calculations with background ambient air from Mt. Gwanak as a reference. The corrected isotope values and the corresponding ambient reference values are summarized in Table 2. Distinct isotopic signatures were observed among the fuel types, whereas no systematic variability was found within the individual fuel categories. Therefore, the mean isotope values were used to represent gasoline, diesel, and CNG exhaust. Gasoline vehicles exhibited δ^13^C of −26.9‰ ± 0.2‰ and δ^18^O of 28.3‰ ± 0.4‰ (1, *n* = 4), diesel vehicles showed δ^13^C of −27.3‰ ± 0.0‰ and δ^18^O of 28.7‰ ± 0.2‰ (1, *n* = 3). In contrast, CNG exhaust exhibited a substantially lower isotopic composition (δ^13^C = −38.8‰; δ^18^O = 25.9‰; 1, *n* = 2).

**TABLE 2:** CO_2_ concentrations and stable isotopic compositions from vehicle emissions and ambient air in this study.

Source	Site description	CO_2_ concentrations	δ^13^C of CO_2_ (‰, VPDB)	δ^18^O of CO_2_ (‰, VSMOW)	*n*
Vehicle (tunnel)	Tunnel (Sangdo)	402.5 ± 222.2 (ppm)	−28.1 ± 0.9	26.9 ± 2.0	4
	Tunnel (Gwanak)	228.2 ± 58.3 (ppm)	−27.4 ± 1.7	25.3 ± 0.9	6
Vehicle (tailpipe)	Gasoline (idling)	3.8 ± 1.8 (%)	−26.7 ± 0.1	28.7 ± 0.0	2
	Gasoline (1500–2000 rpm)	0.6 ± 0.1 (%)	−26.3 ± 0.3	27.1 ± 1.2	2
	Diesel (idling)	7.6 (%)	−27.3	28.5	1
	Diesel (1500–2000 rpm)	6.9 ± 0.3 (%)	−27.3 ± 0.1	28.8 ± 0.2	2
	CNG (idling)	1.9 ± 1.5 (%)	−39.4 ± 0.1	25.8 ± 0.4	2
Ambient air	Mt. Gwanak (629 m a.s.l)	445.2 ± 2.9 (ppm)	−9.6 ± 0.2	40.0 ± 0.1	2

*Note:* All values are mean ± 1 SD, representing the variability among samples. Vehicle (tunnel) values were estimated using mass‐balance calculations based on paired tunnel entrance and exit measurements. Vehicle (tailpipe) values were corrected for ambient air dilution using mass‐balance equations based on Mt. Gwanak ambient air.

### 3.3 N_2_O Stable Isotope Ratios

The stable isotopic ratio of N_2_O in the tunnel air ranged from 4.8 to 6.1 for δ^15^N^bulk^ (‰, Air N_2_), from 42.5 to 43.8 for δ^18^O (‰, VSMOW), and from 16.0 to 18.8 for SP (‰) (Figures 3). For all sampling events, δ^15^N^bulk^, δ^18^O, and SP values were consistently lower at the tunnel exits than at entrances, coinciding with enhanced N_2_O mixing ratios. The ambient air samples collected at the SNU campus and Mt. Gwanak exhibited higher δ^15^N^bulk^, δ^18^O, and SP values than the tunnel air. In addition, tropospheric N_2_O isotopic values were estimated to be approximately δ^15^N^bulk^: 6.3‰ ± 0.2‰ and δ^18^O: 43.9‰ ± 0.3‰ based on extrapolation from Ghosh et al. [56], using the calculated isotopic gradients (Table S6). Keeling plot analysis for the entrance‐exit pairs yielded vehicle‐emitted N_2_O isotopic endmembers of −6.5‰ ± 2.3‰ for δ^15^N^bulk^, 34.1‰ ± 2.2‰ for δ^18^O, and 0.2‰ ± 7.3‰ for SP (1𝜎).

## 4 Discussion

### 4.1 Vehicle Emissions of N_2_O:CO_2_

A moderate positive correlation (*r* ^2^ = 0.44; *p* < 0.05) was observed between ∆CO_2_ and ∆N_2_O measured in the tunnel, suggesting that the two gases are predominantly co‐emitted from vehicles. The N_2_O:CO_2_ emission ratio observed in this study (4.8 ± 1.9 × 10^−2^ ppb:ppm) is higher than those reported in recent tunnel studies conducted in Switzerland and Taiwan [14, 28], yet remains lower than values reported in early studies from the 1990s (Table 1). The lower N_2_O:CO_2_ emission ratios compared to early tunnel studies likely reflect advances in vehicle emission control technologies, including improved three‐way catalyst performance that suppresses N_2_O formation under optimized redox conditions, along with more precise engine control that promotes more complete combustion [21, 29, 30, 59]. However, the observed emission may still be influenced by variations in traffic composition, fuel use (e.g., diesel/LPG ratio), and vehicle age under urban driving conditions [60, 61]. Despite being spatially limited to three tunnels in Seoul, this study directly captures vehicle emissions representative of dense urban on‐road conditions and provides the first measurement‐based estimate of the vehicle N_2_O:CO_2_ emission factor, establishing an important baseline for urban emission inventories.

Applying our observed molar ratio to Seoul's official on‐road transport CO_2_ inventory [32, 62] yields an estimated annual N_2_O emissions of 163 ± 66 kt CO_2_ eq., more than double the current bottom‐up inventory estimate (71 kt CO_2_ eq.). This discrepancy implies that national inventories relying on default Tier 1 emission factors may substantially underestimate vehicular N_2_O contributions, highlighting the need to refine emission factors using real‐world measurements.

Inter‐tunnel differences in N_2_O:CO_2_ emission ratios suggest that traffic composition and fuel type influence this ratio. The Sangdo tunnel, which accommodates a higher proportion of motorcycles and heavy‐duty diesel vehicles, exhibited the lowest mean N_2_O:CO_2_ ratio ([3.9 ± 1.2] × 10^−2^ ppm:ppb, *n* = 16; Table S2). In contrast, the gasoline‐dominated Gwanak and Bongcheon tunnels showed higher mean ratios ([5.2 ± 0.5] × 10^−2^ ppm:ppb, *n* = 13; Figure 2d). Emission factor measurements show that modern gasoline passenger cars generally emit more N_2_O per km than diesel vehicles equipped with after‐treatment systems [63, 64, 65]. Accordingly, the application of on‐road N_2_O:CO_2_ emission ratios requires careful consideration of traffic composition and road characteristics, particularly in urban environments, such as Seoul, where diesel vehicles contribute substantially to total GHG emissions (Table S3).

### 4.2 CO_2_ Stable Isotopes

The tunnel‐derived vehicle‐emitted CO_2_ endmember (δ^13^C: −27.6‰ ± 0.4‰; δ^18^O: 26.8‰ ± 0.5‰) represents an integrated on‐road vehicle emissions isotopic signature in Seoul. The δ^13^C endmember falls within the range reported for gasoline‐ and diesel‐derived CO_2_ in previous studies, confirming that fossil fuel combustion dominates the CO_2_ enhancement observed in tunnel air [8, 50, 51, 52, 53]. The δ^13^C signature in Seoul exhibits a narrow range with minimal variability, reflecting Korea's centralized refinery and fuel distribution system. In contrast, refined gasoline δ^13^C values in Salt Lake City span a much wider range (−26.2‰ to −28.8‰), reflecting the influence of multiple independent refineries processing diverse crude sources [11]. This contrast support the use of the Seoul vehicle‐emitted δ^13^C endmember as a regionally representative constraint for vehicle emissions in East Asian megacities with similarly centralized petroleum refining system.

In contrast to δ^13^C, the δ^18^O endmember reflects both the oxygen source and process‐related modification of vehicle‐emitted CO_2_. Oxygen incorporated during combustion is derived primarily from atmospheric O_2_ (approximately 23.5‰) [9, 18, 66], which is substantially lower than the δ^18^O of background atmospheric CO_2_ values of near 40‰ in the northern mid‐latitudes [16, 54, 55]. The δ^18^O signature can be further modified by isotopic exchange between CO_2_ and water vapor during and after combustion. The δ^18^O signatures observed in traffic‐dominated environments (δ^18^O: 26.8‰ ± 0.5‰) are distinct from those of background atmospheric CO_2_ (40.0‰ ± 0.1‰ at Mt. Gwanak) and reflect process‐specific modifications associated with vehicle emissions.

A fuel‐weighted vehicle CO_2_ isotopic composition (δ^13^C_vehicle_ and δ^18^O_vehicle_) was estimated using fuel‐specific isotopic signatures measured in this study together with independent fleet fuel fractions for Seoul (see Supporting Information Text S1) [50]. The fuel‐weighted δ^13^C_vehicle_ value (−27.8‰ ± 0.1‰) closely agrees with the tunnel‐derived δ^13^C endmember. This agreement demonstrates that δ^13^C provides a robust and fuel‐independent constraint on vehicle‐emitted CO_2_ under real‐world driving conditions. In contrast, the fuel‐weighed δ^18^O_vehicle_ estimate (28.3‰ ± 0.2‰) is approximately 1.5‰ higher than the tunnel‐derived δ^18^O endmember (26.8‰ ± 0.5‰). This offset demonstrates that the post‐emission modification of the integrated exhaust signal is not captured by fuel‐based calculations alone.

### 4.3 N_2_O Stable Isotopes

The lowering of δ^15^N^bulk^, δ^18^O, and SP values observed in tunnel air relative to ambient air at the SNU campus and Mt. Gwanak indicates the addition of ^15^N and ^18^O depleted vehicle‐emitted N_2_O within the tunnel (Figure 3). Vehicle‐emitted N_2_O isotopic compositions showed no substantial differences between the Sangdo and Gwanak tunnels and exhibited limited seasonal variability (Data S2).

**FIGURE 4:**
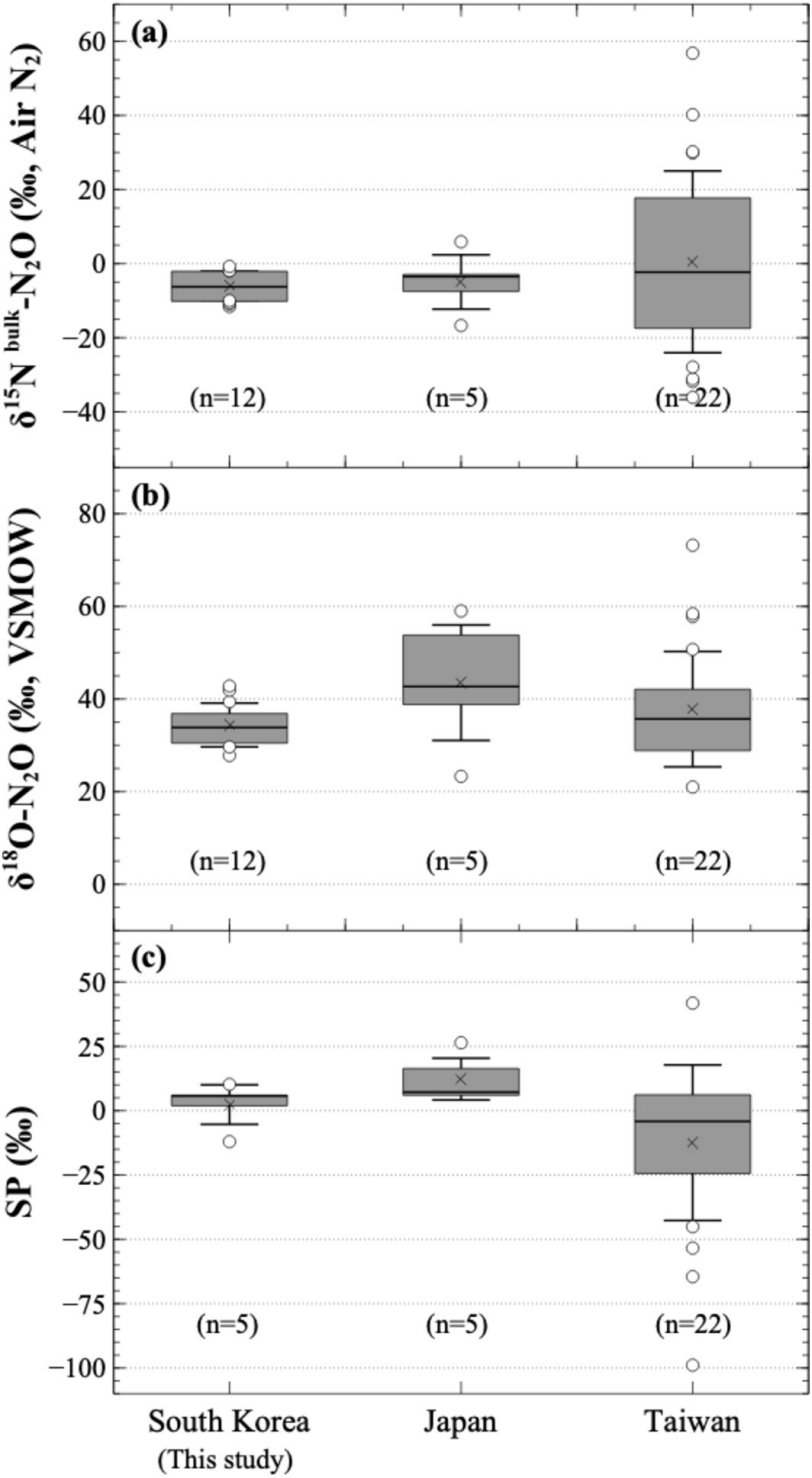
Box plots showing the isotopic composition of N_2_O from vehicles (a: δ^15^N^bulk^, b: δ^18^O, and c: SP). The box represents the one standard deviation range. Japanese data (*n* = 5) are from gasoline passenger vehicles [27], and Taiwanese data (*n* = 22) are from on‐road mixed vehicle fleets [28].

The δ^15^N^bulk^ values of vehicle‐emitted Ν_2_Ο endmembers derived from Keeling plot analysis fall within the range reported for vehicle‐related fossil fuel combustion sources in Japan and Taiwan (Figure 4) [27, 28]. Vehicle‐emitted Ν_2_Ο originates from atmospheric nitrogen (δ^15^N^bulk^≈0‰) and becomes isotopically depleted during high‐temperature combustion due to kinetic isotope effects associated with NOx formation, followed by partial re‐enrichment during exhaust after treatment on three‐way catalysts and SCR systems [67, 68]. The δ^15^N^bulk^ value observed in Seoul (−6.2‰ ± 2.3‰) reflects the integrated effect of these processes and provides a robust, city‐scale constraint for vehicle‐derived Ν_2_Ο source apportionment.

In contrast, δ^18^O of Ν_2_Ο primarily reflects oxygen incorporation pathways during Ν_2_Ο formation and is more sensitive to formation conditions (e.g., catalyst‐mediated oxidation, temperature, and oxygen exchange) than to the isotopic composition of the original oxygen substrates alone [27, 69]. The vehicle‐emitted δ^18^O endmember is consistent with values reported for on‐road vehicle emissions in Japan and Taiwan [27, 28], supporting the use of δ^18^O of Ν_2_Ο in combination with δ^15^N^bulk^ as complementary tracers for constraining both the source and formation processes of vehicle‐derived Ν_2_Ο.

**FIGURE 5:**
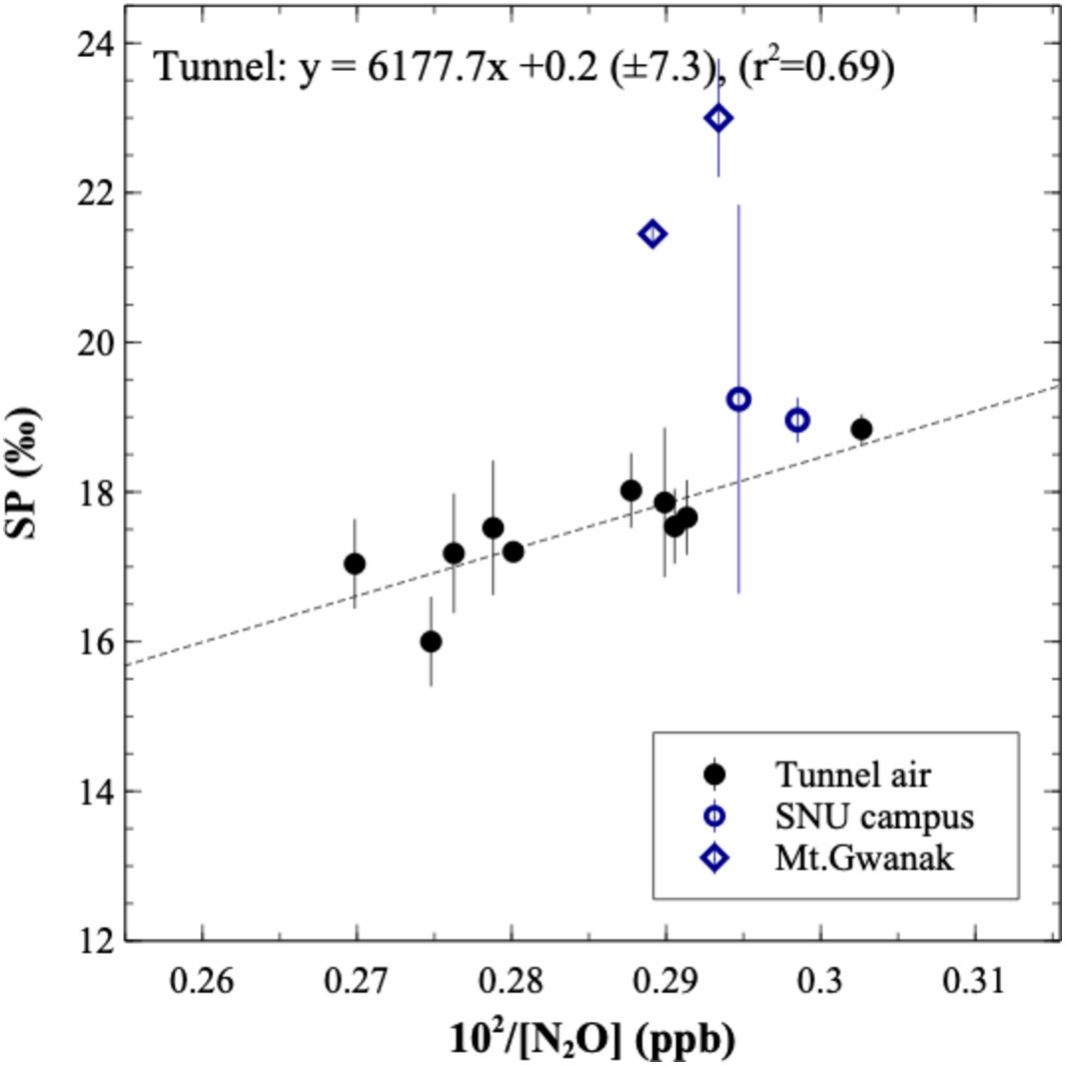
Keeling plots show SP values from the tunnel (black circles) and open atmospheric air from SNU campus (open navy circle) and Mt. Gwanak (open navy diamond).

The SP provides additional process‐level information on vehicle‐emitted Ν_2_Ο formation (Figure 5). Although SP has traditionally been applied to distinguish microbial Ν_2_Ο production pathways, it also serves as a process‐specific tracer for Ν_2_Ο formed during catalytic processes in three‐way converters [59]. Vehicle‐derived Ν_2_Ο from gasoline‐powered vehicles in Seoul exhibits a narrow, near‐zero SP range, reflecting standardized catalyst chemistry and fleet‐averaged driving conditions.

## 5 Conclusions

This study presents a tunnel‐based isotopic characterization of vehicle‐derived CO_2_ and N_2_O emissions in Seoul under real‐world urban driving conditions. The fleet‐averaged vehicular N_2_O:CO_2_ molar ratio ([4.8 ± 1.9] × 10^−2^ ppb:ppm) exceeds the IPCC Tier 1 default by more than a factor of two, indicating that current inventories likely underestimate traffic‐related N_2_O emissions.

Vehicle‐emitted CO_2_ exhibited a narrow δ^13^C signature (−27.6‰ ± 0.4‰) with lower δ^18^O values relative to background air, while vehicle‐emitted N_2_O showed low δ^15^N^bulk^ (−6.5‰ ± 2.3‰) and δ^18^O (34.1‰ ± 2.2‰) values with near‐zero SP (0.2‰ ± 7.3‰), consistent with formation under high‐temperature combustion and catalytic after treatment.

The systematic depletion of oxygen isotopes in both CO_2_ and N_2_O relative to urban background air identifies oxygen isotopes as sensitive tracers of traffic emissions, while the consistency of isotopic signatures across multiple tunnels supports the robustness of the multi‐isotope approach for isolating vehicle‐derived signals in urban environments.

## Author Contributions

**Jeongeun Kim:** conceptualization, methodology, data curation, investigation, validation, formal analysis, visualization, project administration, resources, writing – original draft, writing – review and editing. **Jinho Ahn:** conceptualization, methodology, data curation, investigation, validation, formal analysis, supervision, funding acquisition, project administration, resources, writing – review and editing. **Sambit Ghosh:** methodology, data curation, formal analysis, writing – review and editing. **Sakae Toyoda:** methodology, data curation, writing – review and editing. **Shinji Morimoto:** methodology, data curation, writing – review and editing. **Hyunsuk Choi:** data curation. **Jaehoen Jung:** data curation. **Hungyu Lee:** data curation, writing – review and editing.

## Supporting information




**Figure S1:** (a,b) CO_2_ and N_2_O concentrations from 29 sets of tunnel air samples, showing higher concentrations at the tunnel exits (red dots) compared to the entrances (navy dots) for all three tunnels (Sangdo, Gwanak, and Bongcheon).
**Figure S2:** Plots compare N_2_O stable isotopic results for an internal standard gas from July to November 2021, measured at Seoul National University (SNU, red and navy circle) and Institute of Science Tokyo (Science Tokyo, black and gray circle). Two container types, Silco‐canister (“Can”) and glass flask (“Flask”), were used to assess potential differences due to container type, showing consistent results across both types. Each sample was measured three times at SNU, yielding a final standard error of 0.1‰ for δ^15^N^bulk^ and 0.17‰ for δ^18^O. The results from SNU and the Institute of Science Tokyo showed no significant differences, and the measurements fell within the experimental uncertainty.
**Table S1:** Greenhouse gas emissions from on‐road transportation in Seoul and South Korea in 2021 reported by the Korea Transportation Safety Authority (KOTSA). The greenhouse gas emissions were calculated using Tier 2 and 3 emission factors, following the same methods applied in the Greenhouse Gas Inventory Report of Korea (GIR) (GWP: 310 for N_2_O, 21 for CH_4_).
Note: N_2_O and CH_4_ emissions were excluded from δ^13^C_vehicle_ estimation (account only 1% of total GHG emissions).
**Table S2:** Comparison of *registered vehicle shares by fuel type in Seoul (2021–2024) and **fuel composition of vehicle passing Sangdo Tunnel during peak hours (17:00–19:00). *Registered vehicles data provided by Ministry of Land, Infrastructure, and Transport. **Traffic data for Sangdo Tunnel obtained from the Seoul Institute; values are based on camera monitoring and may include uncertainties. ***HEVs refer to hybrid electric vehicle; electric and hydrogen vehicles are classified under “etc.”
**Table S3:** Greenhouse gas emissions by fuel types for Seoul and South Korea in 2021 reported by the Korea Transportation Safety Authority (CNG, compressed natural gas; HEVs, hybrid electric vehicle; LPG, liquefied petroleum gas).
**Table S4:** N_2_O stable isotopic ratio results found in this study for the tunnel and open atmospheric samples (SNU campus and Mt. Gwanak). Each air samples were measured in triplicate, and the total number of samples is indicated by *n*. The error range between samples represents 1𝝈.
**Table S5:** N_2_O stable isotopic interlaboratory measurement results of samples from Sangdo Tunnel and Seoul National University (SNU) campus which were measured at SNU and Institute of Science Tokyo (Science Tokyo). At SNU, each sample was measured three times while at Science Tokyo, each sample was measured once. The error range for SNU represents the 1𝝈 in triplicate measurements, while for Science Tokyo represents the experimental error of a single sample measurement.
**Table S6:** Global baseline estimates of N_2_O stable isotopic compositions for 2021 derived by extrapolating previously reported global mean values from 2015 using observed long‐term isotopic trends. The global means were obtained from a Monte Carlo inversion incorporating ice core, firn air, and atmospheric observations (Ghosh et al. 2023). Linear regression of isotopic trends over the 1996–2021 period was applied to project baseline values to 2021. Calculation procedures are described in the Supplement Data.
**Text S1: Estimation of δ** ^**13**^ **C**_**vehicle**_.

**Data S1:** Greenhouse gas (CO2 and N2O) concentration and stable isotopic ratio from tunnel, SNU campus, and Mt.Gwanak.
**Data S2:** Vehicle emitted CO2 and N2O stable isotopic ratios by Mass balance equation for each pair (entrance‐exit).
**Data S3:** Monte Carlo estimates of Keeling‐plot enemember uncertainties.
**Data S4:** Data used in N2O isotopic inversion.

## Data Availability

The data that support the findings of this study are available from the corresponding author upon reasonable request.
