# Supplementary material for: CO2 and N2O Emissions From Vehicles in Seoul Megacity, South Korea: Insights From Mixing Ratio and Stable Isotope Ratios
Source: Rapid Commun Mass Spectrom. 2026 Jul 20;40(18):e70094. doi: 10.1002/rcm.70094 (PMC13383353; doi:10.1002/rcm.70094)
Supplement: Supplementary file 1 — Figure S1: (a,b) CO2 and N2O concentrations from 29 sets of tunnel air samples, showing higher concentrations at the tunnel exits (red dots) compared to the entrances (navy dots) for all three tunnels (Sangdo, Gwanak, and Bongcheon). Figure S2: Plots compare N2O stable isotopic results for an internal standard gas from July to November 2021, measured at Seoul National University (SNU, red and navy circle) and Institute of Science Tokyo (Science Tokyo, black and gray circle). Two container types, Silco‐canister (“Can”) and glass flask (“Flask”), were used to assess potential differences due to container type, showing consistent results across both types. Each sample was measured three times at SNU, yielding a final standard error of 0.1‰ for δ15Nbulk and 0.17‰ for δ18O. The results from SNU and the Institute of Science Tokyo showed no significant differences, and the measurements fell within the experimental uncertainty. Table S1: Greenhouse gas emissions from on‐road transportation in Seoul and South Korea in 2021 reported by the Korea Transportation Safety Authority (KOTSA). The greenhouse gas emissions were calculated using Tier 2 and 3 emission factors, following the same methods applied in the Greenhouse Gas Inventory Report of Korea (GIR) (GWP: 310 for N2O, 21 for CH4). Note: N2O and CH4 emissions were excluded from δ13Cvehicle estimation (account only 1% of total GHG emissions). Table S2: Comparison of *registered vehicle shares by fuel type in Seoul (2021–2024) and **fuel composition of vehicle passing Sangdo Tunnel during peak hours (17:00–19:00). *Registered vehicles data provided by Ministry of Land, Infrastructure, and Transport. **Traffic data for Sangdo Tunnel obtained from the Seoul Institute; values are based on camera monitoring and may include uncertainties. ***HEVs refer to hybrid electric vehicle; electric and hydrogen vehicles are classified under “etc.” Table S3: Greenhouse gas emissions by fuel types for Seoul and South Korea in 2021 reported b [file RCM-40-e70094-s001.docx]

**Supporting Information**

**Text S1. Estimation of δ^13^C_vehicle_**

To compare the isotopic composition of CO_2_ directly measured in tunnel air samples with expected values based on fuel usage in Seoul, we calculated fleet-average δ^13^C_vehicle_ and δ^18^O_vehicle_ using greenhouse gas emissions from Korea Transportation Safety Authority (KOTSA, Table S3) and fuel-specific CO_2_ isotopic signatures directly measured from tailpipes. This estimation incorporated three main fuel types: gasoline (δC_g_), diesel (δC_d_), and natural gas (δC_n_). Vehicle fuel types in Seoul include gasoline, diesel, LPG (liquefied petroleum gas), and CNG (compressed natural gas), as well as HEVs (hybrid/electric vehicles). LPG and HEVs were grouped with gasoline: LPG due to its similar δ^13^C values with gasoline (Harris et al., 1999), and HEVs because their internal combustion engine uses gasoline as fuel.

The fraction (*f*) of total CO_2_ emissions contributed by each fuel type in Seoul was determined based on KOTSA data (Table S3). N_2_O and CH_4_ were excluded from the calculation due to their minor contribution (Table S2). The δ^13^C_vehicle_ and δ^18^O_vehicle_ values were calculated using the following equation:

δ^13^C_vehicle_ = (*f*_G+L+H_ × δ^13^C_G_) + (*f*_D_ × δ^13^C_D_) + (*f*_N_ × δ^13^C_N_), (1)

where *f*_G+L+H_, *f*_D_, and *f*_N_ represent the emission fractions of gasoline (including LPG and HEVs), diesel, and natural gas, respectively.

**
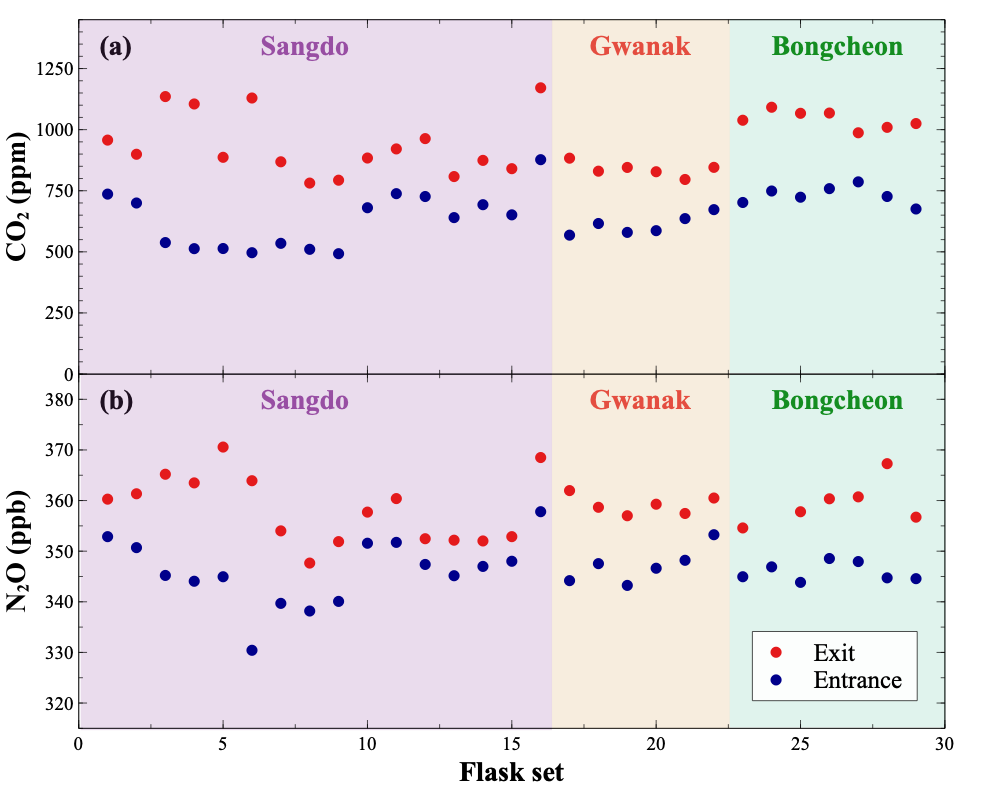
**

**Figure S1.** (a,b) CO_2_ and N_2_O concentrations from 29 sets of tunnel air samples, showing higher concentrations at the tunnel exits (red dots) compared to the entrances (navy dots) for all three tunnels (Sangdo, Gwanak, and Bongcheon).


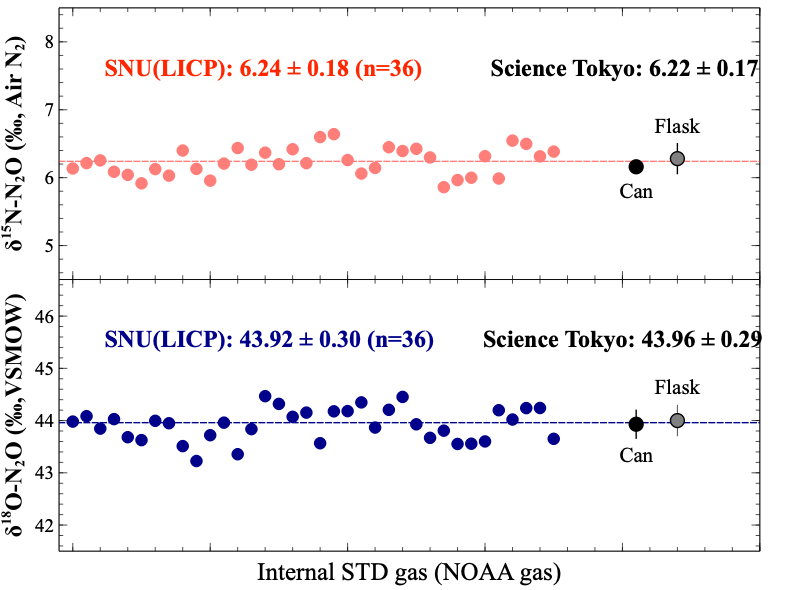


**Figure S2**. Plots compare N_2_O stable isotopic results for an internal standard gas from July to November 2021, measured at Seoul National University (SNU, red and navy circle) and Institute of Science Tokyo (Science Tokyo, black and gray circle). Two container types, Silco-canister (“Can”) and glass flask (“Flask”), were used to assess potential differences due to container type, showing consistent results across both types. Each sample was measured three times at SNU, yielding a final standard error of 0.1‰ for δ^15^N^bulk^ and 0.17‰ for δ^18^O. The results from SNU and the Institute of Science Tokyo showed no significant differences, and the measurements fell within the experimental uncertainty

**Table S1**. Greenhouse gas emissions from on-road transportation in Seoul and South Korea in 2021 reported by the Korea Transportation Safety Authority (KOTSA). The greenhouse gas emissions were calculated using Tier 2 and 3 emission factors, following the same methods applied in the Greenhouse Gas Inventory Report of Korea (GIR) (GWP: 310 for N_2_O, 21 for CH_4_).

Note: N_2_O and CH_4_ emissions were excluded from δ^13^C_vehicle_ estimation (account only 1% of total GHG emissions)

| (Unit: kt CO_2_ eq.) | **CO_2_** | **N_2_O** | **CH_4_** | **Total** |
| --- | --- | --- | --- | --- |
| Seoul | 11,184 (99.0%) | 71 (0.6%) | 41 (0.4%) | 11,296 |
| South Korea | 81,703 | 516 | 120 | 82,339 |

**Table S2.** Comparison of *registered vehicle shares by fuel type in Seoul (2021-2024) and **fuel composition of vehicle passing Sangdo Tunnel during peak hours (17:00-19:00)

*Registered vehicles data provided by Ministry of Land, Infrastructure, and Transport

**Traffic data for Sangdo Tunnel obtained from the Seoul Institute; values are based on camera monitoring and may include uncertainties

***HEVs refer to hybrid electric vehicle; electric and hydrogen vehicles are classified under “etc.”

|  | **Gasoline** | **Diesel** | **LPG** | **CNG** | **HEVs** | **etc.** | **Total** |
| --- | --- | --- | --- | --- | --- | --- | --- |
| **Seoul (2021-2024)** | 51.8 % | 32.2 % | 7.2 % | 0.3 % | 6.2 % | 2.3 % | 100 % |
| **Sangdo Tunnel** | 43.4 % | 35.2 % | 13.5 % | 1.3 % | 5.4 % | 1.3 % | 100 % |

**Table S3.** Greenhouse gas emissions by fuel types for Seoul and South Korea in 2021 reported by the Korea Transportation Safety Authority (LPG: Liquefied Petroleum Gas, CNG: Compressed Natural Gas, HEVs: Hybrid Electric Vehicle).

| (Unit: kt CO_2_eq.) | **Gasoline** | **Diesel** | **LPG** | **CNG** | **HEVs** | **Total** |
| --- | --- | --- | --- | --- | --- | --- |
| **Seoul** | 4,005 (35.5%) | 5,326 (47.1%) | 1,025 (9.1%) | 673 (6%) | 268 (2.4%) | 11,297 |
| **South Korea** | 24,045 (29.2%) | 48,489 (58.9%) | 5,662 (6.9) | 5,459 (3%) | 1,680 (2.0%) | 82,335 |

**Table S4.** N_2_O stable isotopic ratio results found in this study for the tunnel and open atmospheric samples (SNU campus and Mt. Gwanak). Each air samples were measured in triplicate, and the total number of samples is indicated by *n*. The error range between samples represents 1𝝈.

| **Site** | **Sampling date** | **Site detail** | **δ^15^N^bulk^**  **(‰, Air N_2_)** | **δ^18^O**  **(‰, VSMOW)** | **SP (‰)** | ***n*** |
| --- | --- | --- | --- | --- | --- | --- |
| Tunnel | 2021-2024 | entrance | 5.6 ± 0.4 | 43.4 ± 0.2 | 18.0 ± 0.5 | 12 |
|  |  | exit | 5.1 ± 0.3 | 43 ± 0.3 | 17.0 ± 0.6 | 12 |
| SNU campus | 2020-2021 | radio observatory | 6.1 ± 0.2 | 43.9 ± 0.1 | 19.1 ± 0.2 | 2 |
| Mt. Gwanak | 30 June, 2023 | summit (620m) | 5.1 | 44.1 | 21.5 | 1 |
|  | 26 December, 2023 |  | 5.2 | 44.7 | 23.0 | 1 |

**Table S5.** N_2_O stable isotopic interlaboratory measurement results of samples from Sangdo Tunnel and Seoul National University (SNU) campus which were measured at SNU and Institute of Science Tokyo (Science Tokyo). At SNU, each sample was measured three times while at Science Tokyo, each sample was measured once. The error range for SNU represents the 1𝝈 in triplicate measurements, while for Science Tokyo represents the experimental error of a single sample measurement.

| **Location** | **Site** | **Experiment** | **δ^15^N^bulk^ (‰, Air N_2_)** | **δ^18^O (‰, VSMOW)** |
| --- | --- | --- | --- | --- |
| Sangdo Tunnel | Exit 1 | SNU(LICP) | 5.2 ± 0.2 | 42.5 ± 0.4 |
|  |  | Science Tokyo | 4.9 ± 0.2 | 42.2 ± 0.2 |
|  | Entry 1 | SNU(LICP) | 5.8 ± 0.2 | 43.6 ± 0.3 |
|  |  | Science Tokyo | 5.9 ± 0.4 | 43.6 ± 0.7 |
|  | Exit 2 | SNU(LICP) | 5.0 ± 0.1 | 43.0 ± 0.3 |
|  |  | Science Tokyo | 4.6 ± 0.1 | 43.0 |
|  | Entry 2 | SNU(LICP) | 5.7 ± 0.1 | 43.8 ± 0.4 |
|  |  | Science Tokyo | 5.8 ± 0.1 | 44.3 ± 0.5 |
| SNU campus | Obs 1 | SNU(LICP) | 6.2 ± 0.1 | 43.9 ± 0.1 |
|  |  | Science Tokyo | 5.9 ± 0.1 | 44.2 ± 0.1 |
|  | Obs 2 | SNU(LICP) | 6.1 ± 0.1 | 44.0 ± 0.2 |
|  |  | Science Tokyo | 5.5 ± 0.2 | 44.0 ± 0.1 |

**Table S6**. Global baseline estimates of N_2_O stable isotopic compositions for 2021 derived by extrapolating previously reported global mean values from 2015 using observed long-term isotopic trends. The global means were obtained from a Monte Carlo inversion incorporating ice core, firn air, and atmospheric observations (Ghosh et al., 2023). Linear regression of isotopic trends over the 1996-2021 period was applied to project baseline values to 2021. Calculation procedures are described in the Supplement data.

| **Site/method** | **Year** | **δ^15^N^bulk^** | | **δ^18^O** | | **SP** | | **Reference** |
| --- | --- | --- | --- | --- | --- | --- | --- | --- |
|  |  | **‰** | **Std. error** | **‰** | **Std. error** | **‰** | **Std. error** |  |
| Obs_Styx/ | 2015 | 6.6 | 0.1 | 44.1 | 0.1 | 18.5 | 0.4 | Ghosh et al. (2023) |
| Monte Carlo inversion |  | 6.5 | 0.2 | 44.0 | 0.3 | 18.3 | 0.5 |  |
| Global/ gradients for P3 (1966-2021 CE) | 2021 | 6.3 | 0.2 | 43.9 | 0.3 | 18.3 | 0.5 | Calculated with data from Ghosh et al. (2023) |
